# Immunotherapeutic prospects and progress in bladder cancer

**DOI:** 10.1111/jcmm.18101

**Published:** 2024-01-02

**Authors:** Junwei Liu, Yue Gao, Chao Song, Wenbiao Liao, Lingchao Meng, Sixing Yang, Yunhe Xiong

**Affiliations:** ^1^ Department of Urology Renmin Hospital of Wuhan University Wuhan Hubei Province China; ^2^ Department of Party and Administration Renmin Hospital of Wuhan University Wuhan Hubei Province China

**Keywords:** bacillus Calmette‐Guerin, bladder cancer, immune checkpoint inhibitors, immunotherapy

## Abstract

Bladder cancer is one of the most common malignant tumours of the urogenital system, with high morbidity and mortality. In most cases, surgery is considered the first choice of treatment, followed by adjuvant chemotherapy. However, the 5‐year recurrence rate is still as high as 65% in patients with non‐invasive or in situ tumours and up to 73% in patients with slightly more advanced disease at initial diagnosis. Various treatment methods for bladder cancer have been developed, and hundreds of new immunotherapies are being tested. To date, only a small percentage of people have had success with new treatments, though studies have suggested that the combination of immunotherapy with other therapies improves treatment efficiency and positive outcomes for individuals, with great hopes for the future. In this article, we summarize the origins, therapeutic mechanisms and current status of research on immunotherapeutic agents for bladder cancer.

## 1 INTRODUCTION

Bladder cancer is the most common malignant tumour of the urogenital system and has the sixth highest incidence among human cancers.^1^ Bladder cancer is categorized into muscle‐invasive bladder cancer (MIBC) and non‐muscle invasive (superficial) bladder cancer (NMBIC) according to whether it involves muscle invasion. The most common treatments available mainly include surgery, chemotherapy and radiotherapy. Previous research has shown that recurrence is found in 50% of MIBC patients at 2 years after surgery and that 10%–15% of patients have metastases at the time of diagnosis of relapse.^2^, ^3^ Neoadjuvant and adjuvant oncology chemotherapy is commonly used in patients with high‐risk bladder cancer, but approximately 30% of patients have complications following radical cystectomy and are unable to receive adjuvant chemotherapy.^4^ The median 5‐year survival rate of elderly patients with locally advanced or metastatic urothelial cancer on a cisplatin‐based combination chemotherapy regimen is approximately 15%.^5^, ^6^ For patients with MIBC, the overall survival rates at 5 and 10 years after therapy are approximately 50% and 36%, respectively. For the past two decades, these rates have changed substantially due to the development of tumour immunotherapy. For patients who cannot tolerate adjuvant radiotherapy or platinum‐based chemotherapy, especially those at high risk, immunotherapy may be a new approach.^7^, ^8^ Suppression and reprogramming of the immune system play key roles in tumorigenesis and progression, and immunotherapy aims to reactivate antitumor immune cells and overcome the immune escape mechanisms of tumours. Tumour immunotherapy has been called the fourth tumour therapy after surgery, radiation and chemotherapy and was named one of the most valuable scientific breakthroughs in the 21st century.^9^ Tumour immunotherapy strategies include four main categories: nonspecific immune therapy, immune checkpoint inhibitor (ICI) therapy, immunogene therapy and adoptive cell‐transfer therapy. In this paper, we review various types of immunotherapeutic agents currently used that may have therapeutic potential for bladder cancer, not only as monotherapy but also in combination with chemotherapy or other immunotherapeutic agents.

## 2 BACILLUS CALMETTE‐GUERIN

Bacillus Calmette‐Guerin (BCG) is the most common nonspecific immune therapy for bladder cancer. It is a kind of live attenuated strain of *Mycobacterium bovis* that was first reported in 1976 by Morales et al.^10^ for perfusion treatment of non‐muscle invasive carcinoma of the bladder, with a complete remission rate of 70%–80%. The mechanism of action of BCG is that the bacterial wall adheres to uroepithelial cells and bladder tumour cells by interacting with fibronectin, killing cancer cells directly; moreover, it initiates local intrinsic and specific immune responses in bladder tissue, killing cancer cells via immune‐mediated cytotoxicity.^11^ BCG enters tumour cells through phagocytosis to stimulate the production of cellular immune factors such as MCP‐1, MDC, TNF and IL‐10, resulting in the weakening of tumour cell differentiation or stopping the replication cycle. The European Association of Urology (EAU), American Urological Association (AUA) and Chinese Urology Guidelines have listed bladder perfusion with BCG as the standard treatment for intermediate‐to‐high‐risk NMBIC.^12^

At present, the best predictors of response to BCG therapy are clinicopathological features such as tumour grade and stage. Studies have indicated that BCG treatment efficacy and prognosis can be determined by measuring certain immune indicators, such as the number of immune cells, including Treg cells; the ratio of immune molecules in urine or blood; and certain tumour molecular markers, such as P53, survivin, fibroblast growth factor receptor and B‐cell lymphoma/leukaemia‐2.^13^, ^14^ A study by Kamat et al.^15^ detected cytokines such as IL‐1ra, IL‐2, IL‐6, IL‐8, IL‐12, IL‐18, TRAIL, IFN‐γ and TNF‐α in the urine of patients with bladder cancer after BCG infusion, and the accuracy of predicting recurrence was approximately 85.5%.^16^, ^17^ Immune checkpoint inhibitors (ICIs) have largely advanced the progress of bladder cancer immunotherapy research, with the most studied agents being PD‐1, PD‐L1 and CTLA‐4 blocking agents.^18^

## 3 IMMUNE CHECKPOINT INHIBITORS

Immune checkpoints are markers that have a suppressive regulatory role in the immune response. These immune checkpoints, which inhibit T‐cell function under normal conditions, can be used by tumours in tumour‐bearing tissues to create an immune escape effect.^19^ The most widely studied and used immune checkpoints include cytotoxic T‐lymphocyte‐associated antigen‐4 (CTLA‐4), programmed cell death protein‐1 (PD‐1) and programmed death ligand‐1 (PD‐L1). These immune checkpoint inhibitors suppress immune checkpoint activity and reactivate the immune response of T cells against tumours, thus achieving antitumor effects. CTLA‐4 and PD‐1 are receptors expressed on the surface of cytotoxic T cells; CTLA‐4 interacts with the ligand B7, whereas PD‐1 binds the ligand PD‐L1.^20^ These signalling pathways act by assisting tumour cells in evading cytotoxic T‐cell‐mediated death and by preventing receptor and ligand binding, thereby disrupting immunosuppression.^17^

The U.S. FDA has approved five ICIs—atezolizumab, avelumab, durvalumab, nivolumab and pembrolizumab—for treatment of locally advanced and metastatic bladder cancer that has recurred or progressed after first‐line platinum‐based chemotherapy; atezolizumab and pembrolizumab are also approved as first‐line therapies for patients with metastatic bladder cancer who are not candidates for platinum‐based chemotherapy. Overall, ICIs are well tolerated and may be appropriate for fragile patients with impaired renal function (creatinine clearance greater than 30 mL/min), cardiac problems or hearing impairment.^21^ Immune checkpoint inhibitors have emerged as important agents in immunotherapy for advanced bladder cancer with promising applications.

### 3.1 Programmed cell death protein‐1

PD‐1 is mainly expressed on the surface of activated T cells and inhibits the immune response by interacting with the PD‐L1 ligand located on tumour cells. PD‐1 has two ligands—PD‐L1 (B7‐H1, CD274) and PD‐L2 (B7‐DC, CD273)—of which PD‐L2 has an approximately threefold higher affinity for PD‐1 than PD‐L1. However, PD‐L2 expression is limited to the surface of activated dendritic cells (DCs) and some macrophages. By contrast, PD‐L1 is widely expressed on the surface of activated blood cells and epithelial cells, and its expression is induced by type I and II interferons and IL‐10; therefore, most studies have focused on PD‐L1.^22^ After PD‐1 and PD‐L1 bind to activated T cells, they induce dephosphorylation of PD‐1 downstream protein kinases Syk and PI3K, inhibit activation of serine–threonine protein kinase and extracellular signal‐regulated kinase, and finally inhibit transcription and translation of genes and cytokines needed for T‐cell activation, thus playing a negative role in regulating T‐cell activity. Under normal conditions, PD‐1 and PD‐L1 binding induces apoptosis in activated lymphocytes and plays an important role in autoimmune tolerance. A large number of PD‐L1 molecules on the surface of multiple tumour cells bind to PD‐1 molecules on the surface of tumour‐infiltrating lymphocytes and inhibit lymphocyte function, thereby avoiding immune surveillance and leading to tumour immune escape and tumour growth.^23^

Pembrolizumab, an anti‐PD‐1 antibody that blocks binding of PD‐1 to PD‐L1, was used in a large, international, randomized phase III clinical trial, KEYNOTE‐045 (NCT02256436).^24^ In this trial, 542 patients with advanced uroepithelial cancer who developed recurrence or progression after platinum‐based chemotherapy were randomly assigned to receive pembrolizumab or chemotherapy. The median OS was 10.3 months in the pembrolizumab group and 7.4 months in the chemotherapy group, with 12‐month OS rates of 44.4% and 30.2% and 18‐month OS rates of 36.1% and 20.5%, respectively. The overall response rate (ORR) in the pembrolizumab group was significantly higher than that in the chemotherapy group, and the sustained remission rate after 12 months was also significantly higher in the pembrolizumab group. Pembrolizumab is the only immune checkpoint inhibitor with an OS superior to chemotherapy that has been validated in a large, randomized phase III clinical trial. Therefore, it has the highest level of evidence for its effectiveness in treating platinum‐refractory advanced bladder cancer. Jason et al.^25^ reported that combined stereotactic body radiation therapy with pembrolizumab treatment against metastatic solid tumours resulted in an overall objective remission rate of 13.2%, a median OS of 9.6 months (95% CI, 6.5 months to unresolved), and a median progression‐free survival (PFS) of 3.1 months (95% CI, 2.9–3.4 months). Pembrolizumab is an option for high‐risk BCG‐refractory patients who are ineligible for or refuse to undergo cystectomy.

Nivolumab, an anti‐PD‐1 monoclonal antibody, is being studied in a multicenter phase II clinical trial (NCT03519256) for the treatment of high‐risk BCG‐nonresponsive NMIBC. In a phase I and II clinical study (CheckMate032),^26^ 78 patients with metastatic uroepithelial cancer who did not respond to platinum‐based therapy were treated with nivolumab. The median OS was 9.7 months and the median PFS was 2.8 months by the data cutoff, while the 1‐year OS and PFS were 43% and 21%, respectively. The study demonstrated that no significant correlation was found between PD‐L1 expression and nivolumab efficacy. In the CheckMate 275 trial, grade 3–4 adverse events occurred in approximately 18% of patients; the most common adverse event was grade 3–4 diarrhoea, followed by fatigue (33%), pruritus (29%) and rash (15%).^27^ However, clinical trials have shown some adverse renal effects with the use of PD‐1 inhibitors such as nivolumab and pembrolizumab.^28^ Recent phase I/II clinical studies reported a 6.7% incidence of acute kidney injury (AKI) in the form of nephritis. PD‐L1 inhibitors have relatively better tolerance and no propensity for kidney injury and therefore are expected to be a viable alternative for many patients.^29^

### 3.2 Programmed death ligand‐1

PD‐L1 is a membrane surface molecule produced by tumour cells, and interaction of PD‐1 molecules on the surface of T cells with the ligand PD‐L1 initiates a negative regulatory inhibitory signal that inhibits the function of T cells and prevents them from killing tumour cells.^30^

Atezolizumab is a high‐affinity human monoclonal anti‐PD‐L1 antibody. It was authorized by the FDA as a major breakthrough drug in oncology treatment in 2014 and was the first immune checkpoint inhibitor approved for bladder cancer treatment.^31^ A phase III clinical study (IMvigor211)^32^ used atezolizumab for treatment of locally progressive or metastatic bladder cancer after failure of platinum‐based chemotherapy. In the trial, 931 patients were randomly assigned to receive atezolizumab (*n* = 467) or chemotherapy (*n* = 464), with ORR as the primary study endpoint and PFS, OS and safety as secondary endpoints. Atezolizumab was not found to be associated with significantly longer overall survival than chemotherapy in patients with platinum‐refractory metastatic uroepithelial carcinoma who overexpressed PD‐L1 (IC2/3). Nevertheless, atezolizumab has a higher safety profile than chemotherapy.^33^

Durvalumab and avelumab are high‐affinity human IgG monoclonal antibodies against PDL1 that can selectively inhibit PD‐1/PD‐L1 interactions. Boyerinas et al.^34^ found that avelumab induces tumour cell lysis in vitro via the antibody‐dependent cellular cytotoxicity pathway, suggesting that other mechanisms of anticancer activity^35^ may exist. However, avelumab has no significant effect on natural immune cells, thus reducing toxic effects. A phase I and II multicenter study^36^ included 191 patients with locally progressive or metastatic bladder cancer who experienced progression after prior platinum‐based chemotherapy, with a reported to benefit from durvalumab treatment. Grade 3–4 treatment‐related adverse events occurred in 6.8% of the patients.

In an open‐label multicenter phase III clinical study of 1032 patients, durvalumab in combination with tremelimumab treatment did not achieve the expected OS value. On the basis of this study, in February 2021, the FDA withdrew durvalumab approval for second‐line immunotherapy for bladder cancer.^37^ In a study by Massard et al.^38^ of 61 patients treated with durvalumab, no cases of objective remission were found in the PD‐L1‐negative expression group, suggesting that durvalumab is only effective in patients with positive PD‐L1 expression.

### 3.3 Cytotoxic T‐lymphocyte‐associated antigen‐4

CTLA‐4 plays an important role as a negative regulator in CD28‐dependent T‐cell‐mediated immune responses, which is another important signalling pathway for immune checkpoints. Under normal conditions, CTLA‐4 is presented by antigen‐presenting cells (APCs) and binds to APCs via interaction between the CD28 molecule on the T‐cell and the B7 molecule on the APC.

CTLA‐4 has a molecular structure similar to that of CD28 and interacts more readily with ligands with the same structure as CD28; therefore, CTLA‐4 binds to the B7 complex instead of CD28 in vivo, thereby suppressing T‐cell function.^39^ After CTLA‐4 binds to APC, it competitively blocks the interaction between CTLA‐4 and B7 molecules, inhibiting T‐cell activity.^40^ Ipilimumab is a first‐generation synthetic monoclonal antibody targeting anti‐CTLA‐4 that was first approved by the FDA for treatment of unresectable or metastatic melanoma. A phase II multicenter clinical study^41^ demonstrated that use of ipilimumab increased patient response rates by 22% and ORR by 68% compared with conventional treatment with gemcitabine in combination with platinum‐based chemotherapy for metastatic uroepithelial carcinoma. Another clinical study^42^ also showed that presurgical application of ipilimumab significantly improved survival rates.

### 3.4 Immune checkpoint inhibitor‐related therapeutic markers

How to determine which immunotherapy method to choose and how to determine the effectiveness of immunotherapy by testing certain indicators in patients is a major challenge in bladder cancer immunotherapy. Biomarkers of the tumour immune microenvironment and intrinsic characteristics of tumour cells, such as PD‐L1 expression, tumour‐infiltrating lymphocyte density (TIL), tumour mutational load and mismatch repair (MMR) defects, correlate with the efficacy of anti‐PD‐1/anti‐PD‐L1 therapy. In addition, the gut microbiota, circulating biomarkers and patient case histories have been found to be valuable predictors. There is no standardized immunohistochemical assay for the ideal predictive marker PD‐L1, and expression in individual biopsies or surgical specimens alone represents only a single fixed time period and not the entire duration of the disease. Recent studies on IFN‐γ have revealed that loss of function of its signalling pathway is a resistance factor to treatment with ICIs, and patients with high IFN‐γ expression are more likely to show an effect of nivolumab treatment than patients with low IFN‐γ expression, which warrants further prospective trial studies.^43^ In the study by Bandini et al., PD‐L1 expression, high tumour mutational burden (TMB) (>15 mutations/Mb), and mutations in DNA repair genes were associated with pathological response to pembrolizumab.^44^ However, none of these biomarkers was associated with response to pembrolizumab, durvalumab and tremelimumab in the ABACUS study or other trials.^45^ In general, establishing a comprehensive assessment framework that incorporates multiple biomarkers is important for understanding the tumour immune landscape and selecting sensitive patients.

### 3.5 Immune checkpoint inhibitors in combination with other therapies

Currently, ICI therapy is mainly applied in two ways. Neoadjuvant therapy before surgery or radiotherapy is used to improve the complete remission rate of patients, thus increasing the possibility of preserving the bladder. The other method is applied after surgery or radiotherapy to prevent tumour recurrence or metastasis, thus prolonging the progression‐free survival and overall survival of patients. Both of these modalities can be used in combination with chemotherapy or radiotherapy to achieve synergistic effects. A prospective phase II study involved the treatment of 51 patients with cT2‐T4aN0 MIBC with nivolumab in combination with gemcitabine and cisplatin. After a median follow‐up of 24 months, 49 patients completed the neoadjuvant therapy with nivolumab in combination with GC, and 34 patients underwent surgical resection, resulting in a clinical complete remission rate of 58.8% in the patients undergoing combination therapy.^46^ Moreover, three bladder‐preserving immunotherapy studies were presented at the 2021 American Society of Clinical Oncology Annual Meeting, showing good results in MIBC bladder‐preserving treatment with pembrolizumab combined with gemcitabine chemotherapy and concurrent radiation and atezolizumab adjuvant therapy.

### 3.6 Immune‐related adverse events

Studies have shown that application of immunosuppressive drugs leads to different grades of immune‐related adverse events (IrAEs), the incidence of which may reach up to 80.2% and may involve almost all organs and systems of the body.^47^, ^48^ IrAEs not only affect the life quality of patient but may even force patients to discontinue treatment, leading to permanent impairment and, in severe cases, death. The cause of IrAEs may be the coexpression of some antigens on the surface of tumour cells and normal cells, resulting in misuse of normal tissues by the immune system while fighting against tumours.^49^ Studies on ICI precursor drugs—including CX‐072, a drug targeting PD‐1, and BMS‐986249, a drug targeting CTLA‐4—have demonstrated their effectiveness in reducing the incidence of IrAEs.^50^ The main principle of precursor drugs is that after entering the tumour, they need to be cleaved by tumour‐associated lytic enzymes for the active ICIs to be released; the absence of such lytic enzymes in normal cells greatly reduces the damage to the organism.^51^

## 4 IMMUNOGENE THERAPY

Immunogene therapy is a treatment method that delivers target genes into cells through vectors by using gene recombination technology, thus enhancing the body's immune response capability against tumour cells. Some of the widely studied therapies include cytokine gene therapy, tumour antigen gene therapy and antisense gene therapy. In recent years, many studies have also applied these methods for treatment of bladder cancer.^52^ Kimura et al.^53^ transfected bladder cancer cells with CD40L using a gene transfer technique to produce an antitumor immune effect. Pagliaro et al.^54^ demonstrated that immunogene therapy can be safely and effectively applied in the treatment of bladder cancer by applying an adenoviral vector‐mediated wildtype P53 gene for bladder perfusion therapy in patients with high‐stage bladder cancer. Ad‐IFN/Syn3 is an adenoviral vector that induces interferon production in the bladder mucosa by carrying the interferon gene to the bladder mucosa, thereby inhibiting the growth and replication of cancer cells. Studies are underway to replace or potentiate BCG infusion therapy with intravesical Ad‐IFN/Syn3 therapy.^55^ However, few products are actually marketed for clinical application, mainly because of the lack of satisfactory clinical efficacy of gene therapy. For immune gene therapy, a suitable vector needs to be identified that can maintain high efficiency of gene transfection while ensuring safety, high long‐term stability, good targeting and easy preparation.

## 5 ADOPTIVE CELL‐TRANSFER THERAPY (ACT)

ACT is an emerging tumour therapy that isolates immune cells from the patient's body and then expands and modifies them in vitro to be infused back into the patient, thus achieving the goal of directly killing tumour cells or stimulating the body's immune response.^56^ Chimeric antigen receptor T‐cell immunotherapy (CAR‐T) is an important part of ACT in bladder cancer therapy. In CAR‐T cells, genetic engineering modification technology is used to induce a patient's T cells to express chimeric antigen receptors that target tumour cells.^57^ By isolating the patient's own T cells and genetically engineering them in vitro, these T cells express CAR molecules on their surface that can specifically recognize and kill tumour cells. At present, there are more than 500 clinical trials related to CAR‐T cells in the clinical registry, among which CAR‐T cells targeting CD19 are the most studied and have been shown to be effective as treatments of leukaemia, non‐Hodgkin's lymphoma and multiple myeloma.^58^, ^59^ The U.S. and European FDA have approved marketing of CAR‐T cell products for treatment of lymphoma.^60^, ^61^ Because this treatment uses the patient's own cells, it significantly reduces the risk of rejection. Phase I and II clinical studies (NCT03185468) evaluating the efficacy and safety of CAR‐T therapy in locally progressive or metastatic bladder cancer have been conducted.

## 6 CONCLUSION

As discussed above, immunotherapies in treatment of bladder cancer are rapidly advancing. These immunotherapeutic drugs have already shown promising results and will undoubtedly change the therapy landscape for bladder cancer in the future. Several studies are currently underway to determine the optimal tolerated dose of newer drugs alone or in combination with existing chemotherapeutic and/or immunotherapeutic treatments. Despite these great advances in immunotherapy for bladder cancer, there remain many clinical challenges that have not yet been overcome. Firstly, the underlying cellular and molecular mechanisms of immunotherapy remain unclear. Secondly, the need to overcome the challenge of immune escape during immunotherapy has not yet been solved. Furthermore, there is a need to identify molecular markers that can effectively predict the efficacy of immunotherapy and to explore immunotherapy strategies with higher specificity and lower side effects.

## AUTHOR CONTRIBUTIONS

**Junwei Liu:** Conceptualization (equal); data curation (equal); writing – original draft (equal); writing – review and editing (equal). **Yue Gao:** Data curation (equal); writing – original draft (equal). **Chao Song:** Conceptualization (equal); data curation (equal); supervision (equal). **Wenbiao Liao:** Investigation (equal); resources (equal). **Lingchao Meng:** Investigation (equal); resources (equal). **Sixing Yang:** Data curation (equal); writing – review and editing (equal). **Yunhe Xiong:** Formal analysis (equal); writing – review and editing (equal).

## FUNDING INFORMATION

This study was supported by The Interdisciplinary Innovative Talents Foundation from Renmin Hospital of Wuhan University (No. JCRCYR‐2022‐017) and the Nature Science Foundation of Hubei Province (Grant No. 2023AFB691).

## CONFLICT OF INTEREST STATEMENT

All authors declare no conflicts of interest in this study.

## Data Availability

Data sharing is not applicable to this article as no new data were created or analysed in this study.
